# Predictors and determinants of albuminuria in people with prediabetes and diabetes based on smoking status: A cross-sectional study using the UK Biobank data

**DOI:** 10.1016/j.eclinm.2022.101544

**Published:** 2022-07-02

**Authors:** Debasish Kar, Aya El-Wazir, Gayathri Delanerolle, Anna Forbes, James P. Sheppard, Mintu Nath, Mark Joy, Nicholas Cole, J. Ranjit Arnold, Andrew Lee, Michael Feher, Melanie J. Davies, Kamlesh Khunti, Simon de Lusignan, Elizabeth Goyder

**Affiliations:** a Nuffield Department of Primary Care Health Sciences, University of Oxford, UK; b School of Health and Related Research, The University of Sheffield, Sheffield, UK; c National Institute for Health and Care Excellence, UK; d NHS England and Improvement, UK; e Department of Infection, Immunity and Cardiovascular Disease, University of Sheffield, Sheffield, UK; f Department of Clinical Chemistry, Chelsea and Westminster Hospital, London, UK; g Leicester Diabetes Research Centre, University of Leicester, Leicester, UK; h Medical Statistics Team, Institute of Applied Health Sciences, University of Aberdeen, Aberdeen, UK; i University Hospitals of Leicester NHS Trust, Leicester, UK; j Renal Unit, Epsom and St. Helier University Hospital NHS Trust, London, UK; k Royal College of General Practitioners, London, UK; l Department of Diabetes, Leicester NIHR Biomedical Research Centre, Leicester, UK; m Department of Cardiovascular Sciences, Leicester NIHR Biomedical Research Centre, Leicester, UK; n Centre of Excellence in Molecular and Cellular Medicine, Suez Canal University, Ismailia, Egypt

**Keywords:** Albuminuria, Prediabetes, Type 2 diabetes, Smoking

## Abstract

**Background:**

Smoking is attributed to both micro- and macrovascular complications at any stage of metabolic deregulation including prediabetes. Current global diabetes prevention programmes appear to be glucocentric, and do not fully acknowledge the ramifications of cardiorenal risk factors in smokers and ex-smokers. A more holistic approach is needed to prevent vascular complications in people with prediabetes and diabetes before and after quitting.

**Methods:**

A cross-sectional study was carried out on participants who agreed to take part in the UK Biobank dataset at the time of their first attendances between March 01, 2006, and December 31, 2010. Those who had their urinary albumin concentration (UAC) data available were included, and those who did not have this data, were excluded. A logistic regression model was fitted to explore the relationship between cardiorenal risk factors and albuminuria in people with prediabetes and diabetes, based on smoking status.

**Findings:**

A total of 502,490 participants were included in the UK Biobank dataset. Of them, 30.4% (*n*=152,896) had their UAC level recorded. Compared with non-smokers, the odds of albuminuria in smokers with prediabetes and diabetes were 1.21 (95% CI 1.05 – 1.39, *p*=0.009), and 1.26 (95% CI 1.10 – 1.44, *p*=0.001), respectively. The odds declined after quitting in both groups, but it was not statistically significant (*p*>0.05). Each unit increase in HbA1c was associated with equivalent increased odds of albuminuria in current and ex-smokers, OR 1.035 (95% CI 1.030 – 1.039, *p*<0.001), and 1.026 (95% CI 1.023 – 1.028, *p* <0.001), respectively. Compared to females, male ex-smokers were at 15% increased odds of albuminuria. In ex-smokers, each unit increase in waist circumference was associated with 1% increased risk of albuminuria. Compared with the least deprived quintiles, the odds of albuminuria in the most deprived quintiles, in current and ex-smokers were identical, OR 1.18 (95% CI 1.04–1.324, *p*=0.010), and 1.19 (95% CI 1.11 – 1.27, *p*<0.001), respectively.

**Interpretation:**

Male smokers are at a higher risk of albuminuria after smoking cessation. Monitoring waist circumference in quitters may identify those who are at a higher risk of albuminuria. Combining smoking cessation intervention in smokers with prediabetes in the current diabetes prevention programmes may offset post-cessation weight gain and reduce the risk of albuminuria.

**Funding:**

University of Sheffield.

**Research in context**

**Evidence before this study**

Diabetes UK estimates that a third of newly diagnosed people with Type 2 Diabetes Mellitus will have already developed a vascular complication. A third of UK adult population are now having prediabetes and may have already developed complications. The cause for early onset of complications remains unexplored. Lifestyle factors and smoking are likely to be associated. A comprehensive search on PubMed (from January 01, 1996, to January 17, 2022) and Medline (from January 1971 to January 17, 2022) showed that there is an association with smoking and cardiorenal risk factors in any stage of metabolic deregulation. The risk of albuminuria is significantly raised in smokers with T2DM. After smoking cessation, it may take up to 10 years for the risk to decline to the level of non-smokers. Smoking cessation can attenuate the risk in people with diabetes but the effect of smoking cessation in people with prediabetes is not known.

**Added value of this study**

This study suggests that the third of people who are presenting with vascular complication at the onset of T2DM may have developed it at the stage of prediabetes. Smoking is an independent predictor for albuminuria in people with both prediabetes and diabetes. Smoking cessation may cause a rise in waist circumference and can be a more reliable indicator for vascular complication. Halting the progression of HbA1c from prediabetes to diabetes range without smoking cessation in smokers with prediabetes may not protect them from adverse vascular outcomes.

**Implications of all the available evidence**

Smokers with prediabetes should be screened for albuminuria and cardiorenal risk factors. They should be supported to quit and remain abstinent for long-term. Future research should explore the duration of abstinence needed for quitters with prediabetes to get their cardiorenal risk declined to the level of non-smokers.

Alt-text: Unlabelled box

## Introduction

Albuminuria is a sensitive marker for both micro and macrovascular complications at all stages of metabolic deregulation including prediabetes.^1^ It indicates leakage of an abnormal concentration of albumin in the urine. In the disease trajectory of the metabolic syndrome, prediabetes and albuminuria are important hallmarks of disease progression.^2^ In the UK, prediabetes is defined as a state of intermediate hyperglycaemia, when an individual's glycosylated haemoglobin (HbA1c) exceeds the normal range but remains below the threshold for the diagnosis of diabetes.^3^ Due to the lack of consensus about the definition of prediabetes, it is difficult to estimate its global prevalence. However, the International Diabetes Federation (IDF) estimates that the global prevalence of impaired glucose tolerance was 7.5% (374 million) in 2019 and is projected to rise to 8% (454 million) by 2030, and to 8.6% (548 million) by 2045.^4^

Although approximately 25% of people with prediabetes are likely to progress to overt diabetes in 3–5 years,^5^ the risk of vascular complications is considerably higher in all of them, irrespective of whether or not they progress to diabetes. A recent Spanish observational study, Progression of Early Subclinical Atherosclerosis (PESA) showed that compared to those with normoglycaemia, the risk of multi-territorial subclinical atherosclerosis in people with prediabetes was almost 2.5-fold [OR 2.47 (95% CI 1.62-3.76)]. Independent determinants of this heightened risk in this group of people were hypertension, dyslipidaemia and smoking.^6^ Likewise, a recent prospective study on people with prediabetes (*n*=46,911) showed that after a median follow-up of 11 years, 13.8% (*n*=6476) developed atherosclerotic cardiovascular disease, chronic kidney disease or heart failure.^7^ Amongst those who suffered the above adverse vascular outcomes, only 12.4% (*n*=802) progressed from prediabetes to diabetes.^7^ These observations suggest that halting the progression of HbA1c from prediabetes to diabetes range, as recommended by the existing diabetes prevention guidelines worldwide, may not be an effective strategy to prevent the rising surge of vascular complications in people with prediabetes.

Albuminuria is associated not only with nephropathy but also with retinopathy, neuropathy, stroke, peripheral vascular disease, and cardiovascular disease.^8^^,^^9^ Therefore, it may be a reliable composite outcome marker for all of the vascular complications associated with both prediabetes and diabetes. Although multiple studies have explored the link between smoking and albuminuria in diabetes,^10^^,^^11^ evidence in people with prediabetes is lacking. Due to the rapid rise in the number of people with prediabetes developing vascular complications, it is important to screen high-risk people, such as smokers with prediabetes for albuminuria.

Due to the increased risk of vascular complications, all the guidelines worldwide prioritise smoking cessation in people with metabolic deregulation, including prediabetes.^12^ However, the rate of smoking cessation remains identical to the general population. A smoker makes an average of 16 attempts before successfully quitting.^13^ One of the major barriers to successful quitting and remaining abstinent long-term is post-cessation weight gain. Multiple studies have shown that post-cessation weight gain can precipitate the transition from prediabetes to diabetes.^14^^,^^15^ Similarly, in people with diabetes, smoking cessation is associated with a transient rise of HbA1c.^16^ Even following smoking cessation, the risk of major adverse cardiovascular events and mortality remains significantly elevated, in people with diabetes for up to 10 years, compared to non-smokers.^17^ How long it takes for the cardiorenal risk factors in people with prediabetes to decline to the level of non-smokers is unknown. Therefore, two important determinants of the benefit of smoking cessation in people with prediabetes and diabetes are managing post-cessation weight gain, and continued abstinence. In current diabetes prevention programmes, these two important factors are not adequately addressed.

In addition to structured exercise, and dietary intervention in diabetes prevention programmes,^18^ the use of pharmacotherapy has shown promise in facilitating weight loss and reversing prediabetes. A recent double-blind randomised controlled trial (*n*=2254) showed that when people with prediabetes were treated with Liraglutide, 66% of the intervention group, compared to 36% in the placebo group, reverted to normoglycaemia. From the baseline, the Liraglutide group lost an average of 6.5 kg in body weight, compared to 2 kg in the placebo group.^19^ Likewise, in the STEP-2 study, in week 68, participants in the once weekly Semaglutide group lost an average of 15.3 kg in bodyweight from the baseline, compared to 2.6 kg in the placebo group.^20^ Based on this evidence, in the UK, the National Institute for Health and Care Excellence (NICE), approved Liraglutide for the treatment of people with prediabetes, to manage their weight, and prevent progression to diabetes.^21^ However, these interventions are not offered to smokers with prediabetes in any of the diabetes prevention programmes, to help them quit smoking and remain abstinent, and manage post-cessation weight gain.

The aims and objectives of this study are to explore the relationship between cardiorenal risk factors such as age, sex, Body Mass Index (BMI) and blood pressure with albuminuria in people with prediabetes and diabetes. A subgroup analysis examined the predictors of albuminuria in current and ex-smokers.

## Methods

### Study cohort and design

This is a retrospective cross-sectional study using the UK Biobank (UKB) data, containing genomic and phenotyping data of 502,490 individuals from the United Kingdom.^22^ The data used for this study were collected from the screening visits of study participants between March 01, 2006, and December 31, 2010. The data were reported in adherence to the CONSORT reporting guidelines. Out of a total of 9.2 million invited individuals aged 40-69 years, living within 25 miles of 22 recruitment centres in England, Wales, and Scotland, approximately 5.5% (*n*=502,490) consented to take part. UK Biobank received ethics approval from the Northwest Multi-centre Research Ethics Committee (MREC). It has also received approval from the National Information Governance Board for Health & Social Care (NIGB). For this study, ethics approval was also granted by the Research Ethics Committee, the School of Health and Related Research, University of Sheffield (Application No 038586, 09/03/2021). For this cross-sectional study, those who consented to have their urine sample measured for UAC were eligible to be included and those who did not consent were excluded. The study is reported in adherence to the STROBE reporting guidelines for cross-sectional studies.

### Selection process and data collection

At enrolment, each participant completed a lifestyle questionnaire, with their socio-demographic data, and whether they were diagnosed with diabetes, hypertension, stroke, and ischaemic heart disease (IHD). At the recruitment centres, qualified research nurses verified all the information, and undertook a physical assessment including anthropometric measurement and vital signs. They also collected blood and urine samples from the participants who agreed to provide their samples for laboratory analysis. Blood samples were analysed for HbA1c (VARIANT II Turbo, Bio-Rad), total and high-density lipoprotein cholesterol (HDL) (AU5400, Beckman Coulter, Brea, California), and urinary albumin concentration (AU5400, Beckman, Coulter).^23^

### Exposure and covariates

The population of interest for this study was people who responded to the question about whether or not they suffer from diabetes in the questionnaire. Those participants who answered “don't know” or “prefer not to answer” were classed as missing. Prediabetes was defined as no self-reported diabetes, and HbA1c between 6% to 6.4% (42–47 mmol/L), and not on any antidiabetic medication. The exact number of people with type 1 and type 2 diabetes was not possible to be identified from the questionnaire. However, the current epidemiological data suggests that at the population level, 90% of people with diabetes have type 2 diabetes, and therefore, this study assumes that it is representative of people with type 2 diabetes. Participants who had a HbA1c in prediabetes range but were on glucose-lowering medication were classed as having diabetes. The exposure of interest was smoking (defined by self-reported questionnaire as current, former, and non-smokers). For deprivation, the Townsend Deprivation Index was used, which was a composite measure of socioeconomic deprivation derived from employment, household crowding, ownership of house and car, and was assigned by UKB, based on the postcodes of participants’ residence. The Townsend Deprivation Index was used to divide the study participants into quintiles where a negative score in the scale suggested less deprived, and a positive score as more deprived. Body Mass Index (BMI) was expressed in kg/m^2^ and banded as underweight <19 kg/m^2^, normal weight 21–25 kg/m^2^, overweight >25 kg/m^2^ but <30 kg/m^2^ and obese >30 kg/m^2^.

### Outcome variables

The primary outcome measure of the study was urinary albumin concentration (UAC). The spot sample of urine collected at baseline from the study participants was analysed for UAC. *The UK Biobank did not present albumin creatinine ratio (ACR) data as a variable. Therefore, for this study, we used UAC as the measure for albuminuria*. We did not distinguish between microalbuminuria and macroalbuminuria. Following the Kidney Disease: Improving Global Outcomes (KDIGO), we defined a UAC value of 20 mg/l or above in the urine spot sample as albuminuria, and a reading less than 20 mg/l as no albuminuria.^24^

### Statistical analysis

For descriptive statistics, categorical variables were expressed in frequency and percentage, whilst continuous variables were expressed in mean and standard deviation (SD) or median and range, depending on the distribution of data. The statistical significance was set at <0.05. Missing data were excluded from statistical analyses. A logistic regression model was fitted in people with prediabetes and diabetes, to elucidate the relationship of cardiorenal risk factors with albuminuria. Further analysis was carried out to explore how the relationship between cardiorenal risk factors and albuminuria is affected in ex-smokers and current smokers. As the UAC values were missing from almost 70% of the study participants, further analysis was carried out based on age and gender to determine whether the missing values were random.. All data analyses were conducted using software of IBM SPSS statistics version 26 and R version 1.4.

### Role of funding source

The funder of the study had no role in study design, data collection, data analysis, data interpretation, or writing of the report. AE, EG and AL had access to the data and verified the data accuracy. DK has all the data used in the analyses and had the final responsibility for the decision to submit for publication.

## Results

### Descriptive analysis

A total of 502,490 individuals were included in the study, of whom 54.4% (*n*=273,375) were female, and 45.6% (*n*=229,114) were male. UAC data were available for 30.4% (*n*=152,896) of the participants. Out of them, 77.4% (*n*=118,286) had no albuminuria, and 23.5% (*n*=34,610) had albuminuria. A total of 90.8% (*n*=456,293) of the study participants answered the question of whether or not they suffer from diabetes. Of them, 91.82% (*n*=418,950) had normoglycaemia, 4.32% (*n*=19,734) had prediabetes, and 3.86% (*n*=17,609) had diabetes. Those who responded as “don't know” or “prefer not to answer” were classed as missing value.. The mean HbA1c of study participants was 36.1 mmol/mol (5.46%). Smoking data were available for 99.41% (n=499,541) of the study participants. Of them, 54.4% (*n*=273,514) were non-smokers, 34.4% (*n*=173,059) were ex-smokers, and 10.5% (*n*=52,977) were current smokers. The prevalence of smokers and ex-smokers in people with prediabetes was 15.2% (*n*=3009), and 37.9% (*n*=7475), respectively. In those with diabetes, the prevalence of smokers and ex-smokers was 12% (*n*=2113), and 41.1% (*n*=7235), respectively. The prevalence of smoking was significantly higher in prediabetes group, compared to normoglycaemia and diabetes groups. The mean age of the study participants was 57±8 years (Table 1). The missing values were further investigated to ensure that they were missing at random. 56.5% of participants who had their UAC values missing were aged <60 years, and 43.5% were ≥ 60 years. Those who had their UAC data, 50.4% were aged <60 years, 49.6% were ≥ 60 years. Similarly, in people who did not have their UAC data, 57% were female and 43% were male. By contrast, those who had their UAC data, 48.4% were female and 51.6% were male. (Supplementary material 1, Tables 4 and 5).

**Table 1:** Baseline characteristics of the study participants.

Variable		Normoglycaemia(*n*=418950)	Prediabetes(*n*=19734)	Diabetes(*n*=17609)	Missing(*n*=46197)	Overall(*n*=502490)	p-value
Age Mean (SD)		56.7 (8.13)	60.7 (6.84)	59.8 (7.24)	57.7 (8.02)	57.0 (8.10)	<0.001
Sex	Female	231179 (55.2%)	9736 (49.3%)	6758 (38.4%)	25702 (55.6%)	273375 (54.4%)	<0.001
	Male	187771 (44.8%)	9998 (50.7%)	10851 (61.6%)	20494 (44.4%)	229114 (45.6%)	
BMI	Underweight	10477 (2.5%)	172 (0.9%)	84 (0.5%)	1006 (2.2%)	11739 (2.3%)	<0.001
	Normal weight	136840 (32.7%)	2635 (13.4%)	1706 (9.7%)	12224 (26.5%)	153405 (30.5%)	
	Overweight	180302 (43.0%)	7466 (37.8%)	5763 (32.7%)	18387 (39.8%)	211918 (42.2%)	
	Obese	89411 (21.3%)	9309 (47.2%)	9858 (56.0%)	13219 (28.6%)	121797 (24.2%)	
Cholesterol (mmol/L) Mean (SD)		5.75 (1.11)	5.39 (1.29)	4.71 (1.18)	5.62 (1.19)	5.69 (1.14)	<0.001
HbA1c	Mean (SD) mmol/L	34.6 (3.31)	43.8 (1.42)	60.8 (14.0)	42.4 (2.20)	36.1 (6.78)	<0.001
	Mean (SD) %	5.31 (0.303)	6.16 (0.130)	7.71 (1.28)	6.03 (0.201)	5.46 (0.620)	
UAC (mg/L) Mean (SD)		11.1 (6.70, 5420)	13.3 (6.70, 6750)	17.6 (6.70, 5600)	11.9 (6.70, 3800)	11.5 (6.70, 6750)	<0.001
SBP (mm of Hg) Mean (SD)		139 (19.6)	145 (19.5)	145 (18.8)	141 (20.0)	140 (19.7)	<0.001
DBP (mm of Hg) Mean (SD)		82.1 (10.7)	83.4 (10.8)	82.5 (10.8)	82.5 (10.8)	82.2 (10.7)	<0.001
Non-smoker		231826 (55.3%)	9086 (46.0%)	8061 (45.8%)	24541 (53.1%)	273514 (54.4%)	P<0.001
Ex-smoker		142861 (34.1%)	7475 (37.9%)	7235 (41.1%)	15479 (33.5%)	173050 (34.4%)	
Current smoker		42348 (10.1%)	3009 (15.2%)	2113 (12.0%)	5507 (11.9%)	52977 (10.5%)	

Data are presented as frequency (percentage) for qualitative variables, as mean (SD) for parametric quantitative variables and as median (min, max) for non-parametric quantitative variables.

### Prevalence of albuminuria depending on smoking and diabetes status

**Figure 1:**
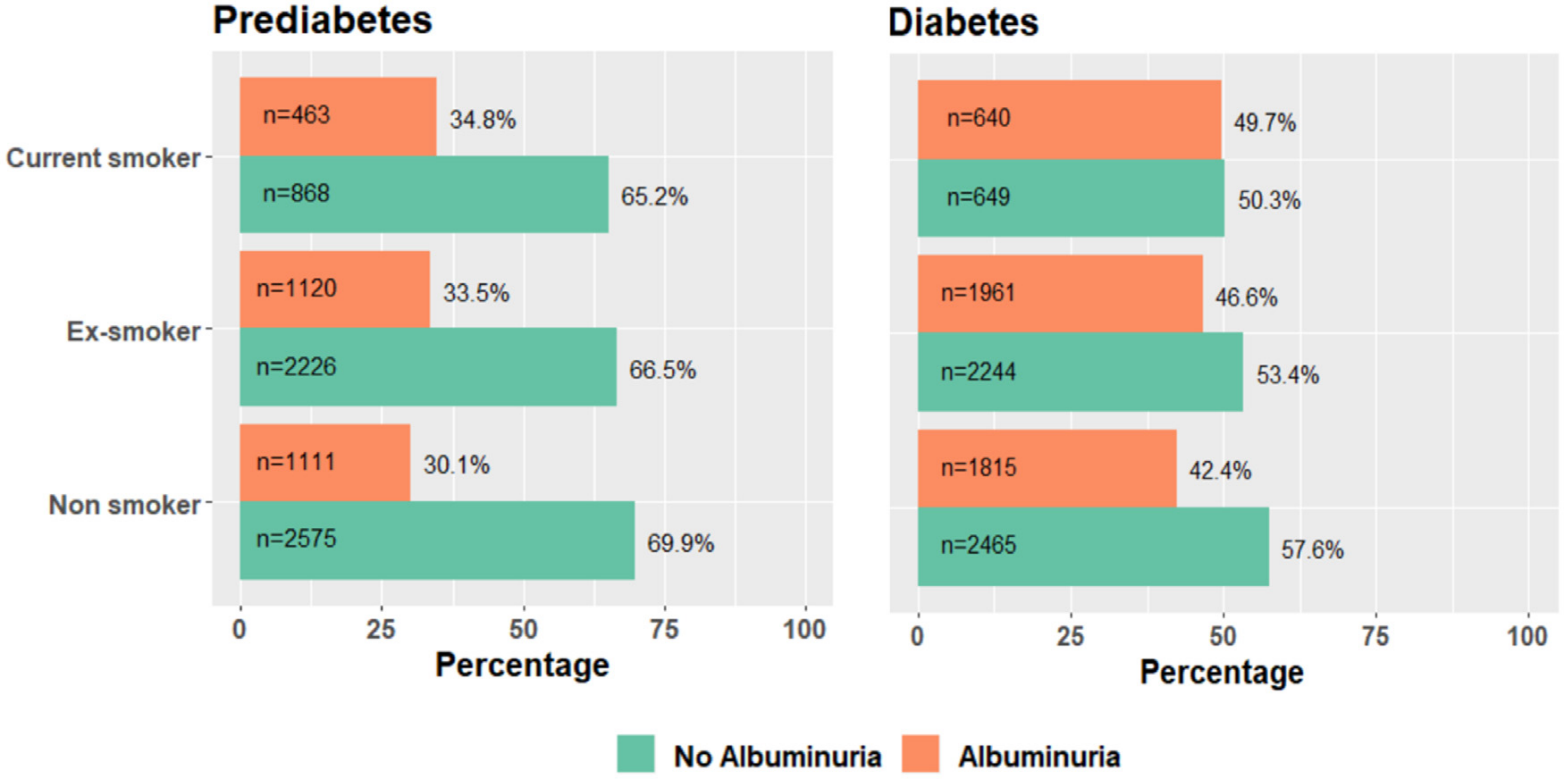
Prevalence of albuminuria in people with prediabetes and diabetes based on smoking status's (Colour codes - Green - no albuminuria, Orange – albuminuria).

Irrespective of smoking status, the prevalence of albuminuria, in people with prediabetes and diabetes were 32.3%, and 45.2%, respectively. Based on smoking status, the prevalence of albuminuria in current, ex and non-smokers with prediabetes were 34.8%, 33.5% and 30.1%, respectively. Similarly, in those with diabetes, the prevalence of albuminuria in current, ex, and non-smokers were 49.7%, 46.6% and 42.4%, respectively (Figure 1).

### Predictors and determinants of albuminuria

We report the estimated odds of albuminuria in people with prediabetes and diabetes based on cardiorenal risk factors such as age, sex, hypertension, HbA1c, cholesterol, ischaemic heart disease (IHD), stroke, deprivation, and BMI.

In people with prediabetes, compared with non-smokers, the adjusted odds ratio of albuminuria in current smokers was 1.21 (95% CI 1.05 – 1.39, p=0.009), and in ex-smokers was 1.07 (95% CI 0.96 – 1.19, *p*=0.240). Other cardiorenal risk factors associated with increased odds of albuminuria were - male sex 1.42 (95% CI 1.28 – 1.58, *p*<0.0001), stroke 1.71 (95% CI 1.30 - 2.25, *p* <0.0001), ischaemic heart disease 1.41 (95% CI 1.20 - 1.65, *p*<0.0001), HbA1c 1.06 (95% CI 1.02 – 1.10, *p*<0.0001), and hypertension 1.57 (95% CI 1.40 - 1.75, *p*<0.0001). Age, cholesterol, deprivation, and BMI did not show any statistically significant relationship with albuminuria at the stage of prediabetes (Table 2).

**Table 2:** Logistic regression model with adjusted estimates of odds ratio and corresponding 95% confidence intervals for predictors of albuminuria at the stage of prediabetes.

Cardiorenal risk factors	Odds ratio (95% CI)	*p*-value	Effect size (S.E)
Current smokers vs non-smoker	1.21 (1.05 – 1.39)	0.009	0.191 (0.072)
Ex-smokers vs non-smokers	1.07 (0.96 – 1.19)	0.240	0.065 (0.055)
Age	0.99 (0.99 – 1.00)	0.536	-0.003 (0.004)
Male sex	1.42 (1.28 – 1.58)	<0.001	0.353 (0.052)
Most deprived quintile vs least deprived quintiles	1.05 (0.90 – 1.22)	0.566	0.045 (0.078)
HbA1c	1.06 (1.02 – 1.10)	0.001	0.058 (0.017)
Cholesterol	1.00 (0.96 – 1.05)	0.838	0.004 (0.021)
Overweight vs normal weight	0.64 (0.38 – 1.07)	0.091	-0.444(0.263)
Obese vs normal weight	0.84 (0.50 – 1.40)	0.500	-0.176 (0.262)
Stroke	1.71 (1.30 – 2.25)	<0.001	0.536 (0.140)
Ischaemic heart disease	1.41 (1.20 – 1.65)	<0.001	0.343 (0.082)
Hypertension	1.57 (1.40 – 1.75)	<0.001	0.450 (0.056)

In people with diabetes, the odds of albuminuria in current smokers were 1.26 (95% CI 1.10 – 1.44, *p*<0.0001), and in ex-smokers 1.09 (95% CI 0.99 – 1.19, *p*=0.08). Other cardiorenal risk factors associated with albuminuria in people with diabetes were - male sex 1.52 (95% CI 1.38 – 1.67, *p*<0.0001), stroke 1.96 (95% CI 1.52 - 2.52, *p*<0.0001), ischaemic heart disease 1.79 (95% CI 1.57 - 2.04, *p*<0.0001), cholesterol 1.06 (95% CI 1.03 – 1.10, *p*<0.0001), HbA1c 1.014 (95% CI 1.011 – 1.018, *p*<0.0001) and hypertension 1.55 (95% CI 1.40 - 1.71, *p*<0.0001). There was no statistically significant relationship between age, and BMI with albuminuria, in people with diabetes (Table 3). In diabetes group, after adjusting for smoking status and other cardiorenal risk factors, those who were in the most deprived quintile, compared with those who were in the least deprived quintiles, were 35% more likely to have albuminuria (Table 3).

**Table 3:** Logistic regression model with adjusted estimates of odds ratio and corresponding 95% confidence intervals for predictors of albuminuria at the stage of diabetes.

Cardiorenal risk factors	Odds ratio (95% CI)	p-value	Effect size (S.E)
Current smokers vs non-smoker	1.26 (1.10 – 1.44)	0.001	0.242 (0.066)
Ex-smokers vs non-smokers	1.09 (0.99 – 1.19)	0.080	0.084 (0.046)
Age	1.00 (0.99 – 1.01)	0.536	0.002 (0.003)
Male sex	1.52 (1.38 – 1.67)	<0.001	0.419 (0.048)
Most deprived quintile vs least deprived quintiles	1.35 (1.18 – 1.55)	<0.001	0.302 (0.071)
HbA1c	1.01 (1.01 – 1.02)	<0.001	0.014 (0.002)
Cholesterol	1.06 (1.03 – 1.10)	0.001	0.062 (0.019)
Overweight vs normal weight	0.65 (0.30 – 1.41)	0.091	-0.435(0.399)
Obese vs normal weight	0.83 (0.38 – 1.82)	0.647	-0.182 (0.398)
Stroke	1.96 (1.52 – 2.52)	<0.001	0.672 (0.128)
Ischaemic heart disease	1.79 (1.57 – 2.04)	<0.001	0.582 (0.068)
Hypertension	1.55 (1.40 – 1.71)	<0.001	0.438 (0.051)

This study shows that in people with diabetes, smoking and deprivation are independently associated with albuminuria. However, the data from the Office for National Statistics (ONS) in the UK showed that smoking prevalence is 4 times higher in the most deprived neighborhoods, compared with the least deprived,^25^ suggesting that there may be an overlapping relationship between smoking, socioeconomic status and albuminuria.

All assumptions of binary logistic regression were tested and confirmed as met, including the presence of a binary dependent variable, independence of observations, absence of correlations between independent variables and absence of significant outliers in numeric independent variables. All continuous independent variables included in the model were linearly related to the log of odds.

### Relationship of smoking status and cardiorenal risk factors with albuminuria

**Figure 2:**
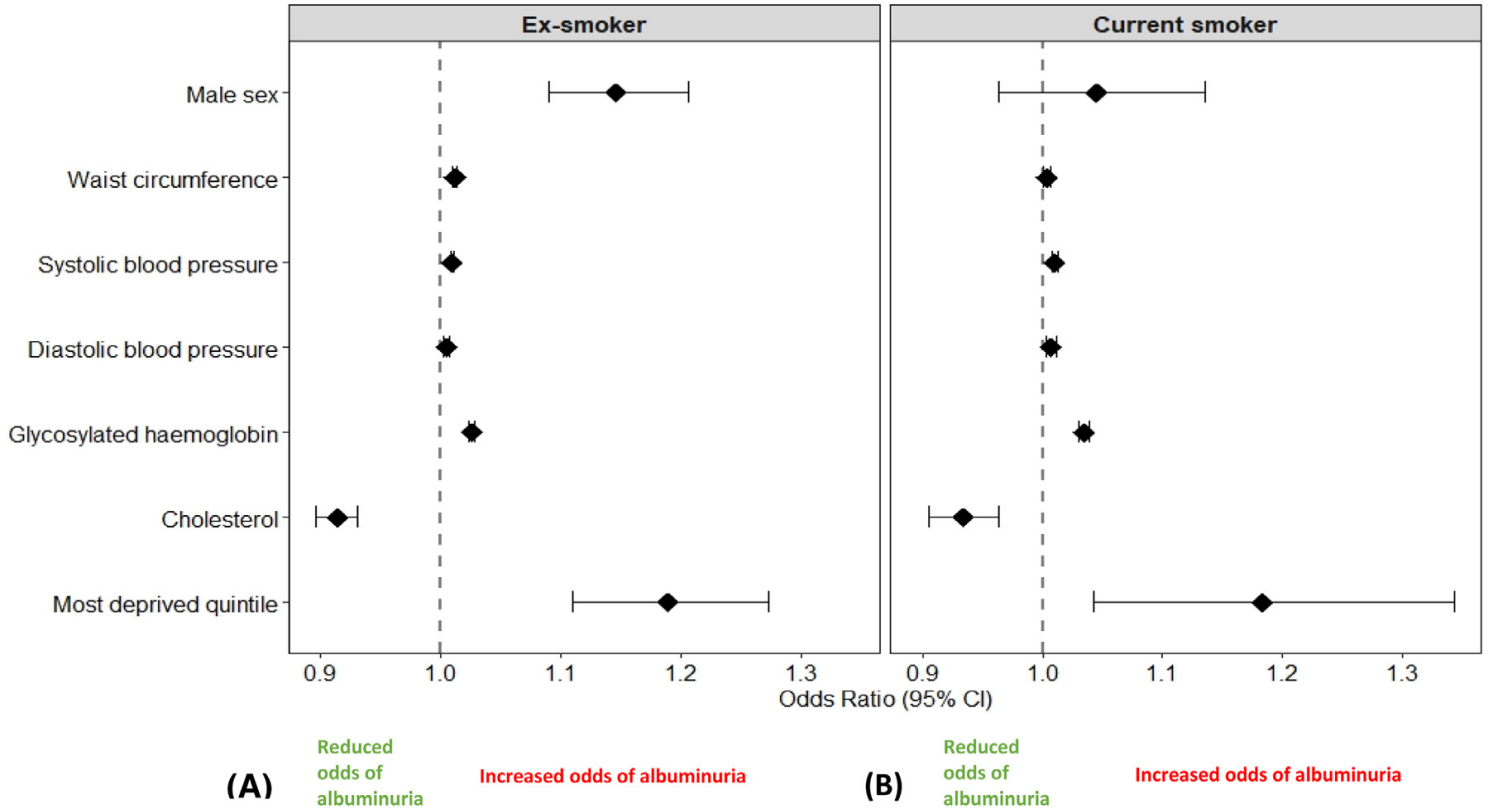
Risk of albuminuria in ex-smokers and current smokers (Red – increased risk of albuminuria – please insert this on the right side of the vertical line of the forest plot).

In ex-smokers, the odds of albuminuria in male sex, systolic and diastolic blood pressure, cholesterol, HbA1c, waist circumference and the most deprived quintiles were 1.146 (95% CI 1.090 – 1.206, *p*<0.001), 1.009 (95% CI 1.008 – 1.011, *p*<0.001), 1.005 (95% CI 1.002 – 1.007, *p*<0.001), 0.914 (95% CI 0.896 – 0.931, *p*<0.001), 1.026 (95% CI 1.023 – 1.028, *p*<0.001), 1.012 (95% CI 1.010 – 1.013, *p*<0.001), and 1.189 (95% CI 1.110 – 1.273, *p*<0.001), respectively. [Figure 2 (A)]

In current smokers, the cardiorenal risk factors associated with increased odds of albuminuria were systolic blood pressure 1.010 (95% CI 1.008 – 1.013, *p*<0.001), diastolic blood pressure 1.007 (95% CI 1.003 – 1.012, *p*=0.001), Cholesterol 0.934 (95% CI 0.905 – 0.964, *p*<0.001), HbA1c 1.035 (95% CI 1.030 – 1.039, *p*<0.001), waist circumference 1.004 (95% CI 1.001 – 1.007, *p*=0.004), and deprivation 1.183 (95% CI 1.042 – 1.343, *p*=0.010). In current smokers, there was no evidence of an association between male sex and albuminuria [OR 1.045 (95% CI 0.963 – 1.135), *p* = 0.292)]. [Figure 2 (B)].

## Discussion

This cross-sectional study demonstrated that smoking is an important risk factor for albuminuria in both people with prediabetes and diabetes. This finding is in keeping with the current evidence.^26^^,^^27^ Irrespective of diabetes status, male ex-smokers are at a higher risk of albuminuria than current smokers. Smoking cessation may not reverse the risk immediately after quitting. One of the key determinants of the risk of albuminuria after quitting is post-cessation weight gain, indicated by waist circumference. This study suggests that waist circumference may be a better predictor of albuminuria than BMI. Deprivation is an independent predictor for albuminuria in both ex- and current smokers. Systolic and diastolic blood pressure, and HbA1c are positively associated with albuminuria in both ex- and current smokers, suggesting that these risk factors should be addressed in both groups. Total cholesterol was negatively associated with albuminuria in both current and ex-smokers.

In people with prediabetes, each unit increase in HbA1c was associated with 6% increased odds of albuminuria, compared with 1% in those with diabetes, suggesting that controlling HbA1c may be more important in people with prediabetes than diabetes. Hypertension, IHD and stroke are independently associated with albuminuria in both groups. In people with diabetes, each unit increase of cholesterol was associated with 6% increased odds of albuminuria, but in those with prediabetes, there was no statistically significant association between total cholesterol and albuminuria. Similarly, there was no evidence that BMI was associated with albuminuria in either group. Compared with the least deprived quintiles, the most deprived quintiles of people in diabetes group had a higher odd of albuminuria, but not in those in the prediabetes group. Compared with non-smokers, despite lower BMI, smokers have larger waist circumference,^28^^,^^29^ which is an independent risk factor for insulin resistance,^30^ and albuminuria.^31^ After smoking cessation, quitters may have an increased waist circumference as well as BMI. Therefore, two important determinants of albuminuria in smokers are avoiding post-cessation weight gain and remaining abstinent long-term.^32^^,^^33^ This is the first study to show that greater waist circumference may also be associated with a higher risk of albuminuria in ex-smokers.

In people with diabetes, previous studies have shown that the progression of nephropathy can be ameliorated in those who gave-up, compared with those who continued to smoke.^34^ However, the impact of smoking and its cessation on albuminuria in people with prediabetes is not fully understood. Anecdotal evidence suggests that the duration of abstinence and post-cessation weight gains could be the two determinant factors of albuminuria in ex-smokers.^35^ This study supports the risk of ex-smokers of albuminuria based on waist circumference, but a longitudinal study will be needed to estimate the length of abstinence needed to bring the risk down to the level of non-smokers. Current Diabetes Prevention Programmes worldwide assume that in people with prediabetes, keeping the HbA1c under the diabetes range will be sufficient to keep the vascular risk under control. However, the findings of this cross-sectional study suggest that it may be a piecemeal approach, particularly for smokers and ex-smokers with prediabetes, who are at a higher risk of albuminuria.

Microalbuminuria is an important milestone in the disease trajectory of metabolic syndrome. If renal disease can be detected at the stage of microalbuminuria, and multifactorial intervention initiated at an early stage of disease trajectory, it can improve the renal outcome and reduce the risk of all-cause and cardiovascular mortality.^36^ Smoking cessation, active weight management, glycemic control, and hypertension managements are the key components of the multifactorial intervention.

However, the above multifactorial intervention approach can only reverse the renal impairment if it can be implemented at the stage of microalbuminuria. When microalbuminuria progresses to proteinuria and a decline in estimated Glomerular Filtration Rate (eGFR) takes place, the renal dysfunction is irreversible, although inhibition of Renin-Angiotensin-Aldosterone System (RAAS) can slow down the progression.^37^

Hypertension management in smokers and ex-smokers with prediabetes and diabetes can be challenging and needs careful monitoring. Although RAAS inhibitors have dual effects to lower blood pressure and to prevent albuminuria, their effectiveness is diminished in smokers, due to enhanced pulmonary conversion of angiotensinogen to angiotensin II, which has a potent vasoconstrictor property.^38^ Similarly, the effectiveness of beta-blockers to treat hypertension in smokers is counteracted by the direct excitatory effect of nicotine on sympathetic adrenergic pathway.^39^ Therefore, without smoking cessation, effective blood pressure control may be difficult to achieve.

In contrast to the current understanding,^40^ this study found that cholesterol had a negative relationship with albuminuria in both ex-smokers and current smokers. The precise cause for this observation is unclear. One of the explanations could be that due to the higher socio-economic status and education level, the UKB population may have a healthier diet which increased the level of HDL-cholesterol, than triglycerides and LDL cholesterol. Previous studies have shown that higher HDL concentration can reduce the risk,^41^ while high LDL,^42^ and triglyceride^43^ can increase the risk of albuminuria.

The findings of this study are important for public health policy. The risk of albuminuria is high in smokers and ex-smokers with prediabetes. Current diabetes prevention models, without smoking cessation intervention, and without screening for vascular complications such as albuminuria, may lead to a late presentation, with established microvascular and macrovascular complications, when they are not amenable to reversal. Smokers with prediabetes and diabetes should be supported not only to quit smoking, but also to remain abstinent. Short-term quitting may not give any meaningful risk reduction benefit.

The main strengths of this study are its sample size and novel stratified analysis. This is the first study to demonstrate the risk of albuminuria, in smokers and ex-smokers with prediabetes. It identifies the gaps in the current diabetes prevention models and how the weight loss gained from the structured lifestyle intervention can be used opportunistically to encourage smokers to quit and remain abstinent. It also demonstrates that a holistic, multifactorial approach is needed to reduce the risk of albuminuria in people with prediabetes rather than just keeping the HbA1c below diabetes range.

However, there are several limitations associated with the dataset that makes the interpretation of the findings less generalizable. Instead of ACR, we used the available UAC data, albeit it was only for 30% of the study participants. We do not hold data about the other 70% of the UK Biobank population to know if there was selection bias; however, we do know that those in the UK Biobank cohort overall are more likely to be of white ethnicity and higher socioeconomic status.^22^

We did not differentiate micro and macroalbuminuria, and therefore, unable to comment on how the cardiorenal risk factors influence the outcomes in current and ex-smokers. From the questionnaire, it was not possible to be precise about the numbers of type 1 and type 2 people with diabetes which is one of the major weaknesses of this study. We recognise that a single measure of albuminuria does not enable us to assess variability, which in itself is a risk factor in both type 1 and type 2 diabetes.^44^^,^^45^

The age of the study participants was limited to 40-69 years. People from the Black, Asian, and Minority Ethnicities (BAME) were not well-represented, 94% of the study participants being from the White ethnicity. In the UKB data, the prevalence of smoking was 10.5%, while the ONS data 2020 suggests that at population level, in the UK, the prevalence of smoking in adults aged 16 and above is 14.5%.^46^ Therefore, the risk of albuminuria in smokers might have been underestimated in this study. We also note that the prevalence of smoking was lower in the UKB population than the national population, possibly reflecting selection bias, related to its higher socioeconomic status.^22^ The smoking data was derived from the questionnaire and no biochemical confirmation was sought. This is a less reliable method of determining smoking status than biochemical affirmation by measuring urinary excretion of nicotine metabolites.

The cross-sectional design of the study could not elucidate how long the quitters need to remain abstinent for their risk to decline to the level of non-smokers. The UKB study participants were less likely to be socioeconomically deprived, obese and smokers than the general population. They were less likely to drink alcohol daily, had fewer self-reported physical and/or mental health conditions, and were more likely to be older and female. Cross-sectional study design is a limitation as it cannot establish causation. However, it serves the purpose of hypothesis generation and helps inform the design of future prospective studies.

In this study, we demonstrated there was no evidence that smoking cessation reduced the risk, suggesting that quitters may need to remain abstinent for a length of time, but the precise length could not be established. A prospective study is needed to answer this question. Similarly, the effectiveness of using GLP-1 analogues (i.e., Liraglutide, Semaglutide) in managing post-cessation weight gain need to be explored. A randomized controlled trial is needed to investigate the efficacy of GLP-1 analogue to prevent weight gain in ex-smokers and facilitate long-term abstinence. A longitudinal study is required to elucidate how long the ex-smokers need to remain abstinent to get their risk declined to the level of non-smokers.

## Contributors

This is a part of the MD project of lead author DK. He had the concept, and was responsible for study design, writing the protocol for data access, applying for ethical approval, conducting the statistical analyses, and writing up the manuscript. AE was involved in the data cleaning, and statistical analyses. MN and MJ checked the statistical analyses. EG, AL and SdeL worked in supervisory capacities. All other co-authors read the manuscript and gave their feedback. AE, EG and AL had full access and verified the data. The lead author DK has full access to all the data in the study and had final responsibility for the decision to submit for publication.

## Data sharing statement

The genetic and phenotypic UK Biobank data are available on application to the UK Biobank to any researcher worldwide (www.ukbiobank.ac.uk). This is an open access article distributed with the Creative Commons Attribution (CC By 4.0) license, which permits others to copy, redistribute, remix, transform and build upon this work for any purpose, provided the original work is properly cited, a link to the license is given, and indication of whether changes were made. (https://creativecommons.org/licenses/by/4.0/)

## Declaration of interests

JPS reports funding from the Wellcome Trust/Royal Society via a Sir Henry Dale Fellowship (ref: 211182/Z/18/Z) and an NIHR Oxford Biomedical Research Centre (BRC) Senior Fellowship; grants from the British Heart Foundation and Stroke Association; consulting fees from Doctorlink; and participation on a data safety monitoring board/advisory board for the hypertension in Nigeria trial (NCT04158154). JRA is supported by a NIHR Clinician Scientist Award (CS 2018-18-ST2-007). SdeL reports that through his university, he has had grants not directly relating to this work, from AstraZeneca, GSK, Sanofi, Seqirus, and Takeda for vaccine research and membership of advisory boards for AstraZeneca, Sanofi and Seqirus. KK is supported by the National Institute for Health Research (NIHR) Applied Research Collaboration East Midlands (ARC EM) and the NIHR Leicester Biomedical Research Centre (BRC). KK acted as a consultant and speaker for Amgen, AstraZeneca, Bayer, Novartis, Novo Nordisk, Roche, Sanofi-Aventis, Lilly, Servier and Merck Sharp & Dohme. He has received grants in support of investigator and investigator-initiated trials from AstraZeneca, Novartis, Novo Nordisk, Sanofi-Aventis, Lilly, Pfizer, Boehringer Ingelheim and Merck Sharp & Dohme. KK has received funds for research, honoraria for speaking at meetings and has served on advisory boards for AstraZeneca, Lilly, Sanofi-cool Aventis, Merck Sharp & Dohme, and Novo Nordisk. MJD reports grants from AstraZeneca, Boehringer Ingelheim, Sanofi, Novo Nordisk, and Janssen; consulting fees from Novo Nordisk, Eli Lilly, Sanofi, and Boehringer Ingelheim; payments from Novo Nordisk, Eli Lilly, AstraZeneca, Takeda Pharmaceuticals International, Sanofi, Boehringer Ingelhiem, and Napp Pharmaceuticals; and participation on a data safety monitoring board or advisory board for Novo Nordisk, Eli Lilly, AstraZeneca, Janssen, Sanofi, Boehringer Ingelhiem, Gilead Sciences, and Lexicon. DK, EG, AL, GD, AF, AE, MN, NC, MF, and MJ declare no conflict of interest.

## Appendix Supplementary materials

Image, application 1

Image, application 2

## References

[1] Deckert T, Kofoed-Enevoldsen A, Norgaard K, Borch-Johnsen K, Feldt-Rasmussen B, Microalbuminuria JT (1992). Implications for micro- and macrovascular disease. Diabetes Care.

[2] Brannick B, Wynn A, Dagogo-Jack S (2016). Prediabetes as a toxic environment for the initiation of microvascular and macrovascular complications. Exp Biol Med.

[3] Bansal N (2015). Prediabetes diagnosis and treatment: a review. World J Diabetes.

[4] Saeedi P, Petersohn I, Salpea P (2019). Global and regional diabetes prevalence estimates for 2019 and projections for 2030 and 2045: results from the international diabetes federation diabetes atlas, 9. Diabetes Res Clin Pract.

[5] Tabák AG, Herder C, Rathmann W, Brunner EJ, Kivimäki M (2012). Prediabetes: a high-risk state for diabetes development. Lancet North Am Ed.

[6] Rossello X, Raposeiras-Roubin S, Oliva B (2021). Glycated hemoglobin and subclinical atherosclerosis in people without diabetes. J Am Coll Cardiol.

[7] Honigberg MC, Zekavat SM, Pirruccello JP, Natarajan P, Vaduganathan M (2021). Cardiovascular and kidney outcomes across the glycemic spectrum. J Am Coll Cardiol.

[8] Savage S, Estacio RO, Jeffers B, Schrier RW (1996). Urinary albumin excretion as a predictor of diabetic retinopathy, neuropathy, and cardiovascular disease in NIDDM. Diabetes Care.

[9] Mohammedi K, Woodward M, Hirakawa Y (2016). Microvascular and macrovascular disease and risk for major peripheral arterial disease in patients with type 2 diabetes. Diabetes Care.

[10] Xu H, Suo J, Lian J (2018). Cigarette smoking and risk of albuminuria in patients with type 2 diabetes: a systematic review and meta- analysis of observational studies. Int Urol Nephrol.

[11] Harmer JA, Keech AC, Veillard A-S (2014). Cigarette smoking and albuminuria are associated with impaired arterial smooth muscle function in patients with type 2 diabetes mellitus: a FIELD substudy. Diabetes Res Clin Pract.

[12] David L (2020). 2019 ESC Guidelines on diabetes, pre-diabetes, and cardiovascular diseases developed in collaboration with the EASD. Eur Heart J.

[13] Chaiton M, Diemert L, Cohen JE (2016). Estimating the number of quit attempts it takes to quit smoking successfully in a longitudinal cohort of smokers. BMJ Open.

[14] Pan A, Wang Y, Talaei M, Hu FB, Wu T (2015). Relation of active, passive, and quitting smoking with incident type 2 diabetes: a systematic review and meta-analysis. Lancet Diabetes Endocrinol.

[15] Hu Y, Zong G, Liu G (2018). Smoking cessation, weight change, type 2 diabetes, and mortality. N Engl J Med.

[16] Lycett D, Nichols L, Ryan R (2015). The association between smoking cessation and glycaemic control in patients with type 2 diabetes: a thin database cohort study. Lancet Diabetes Endocrinol.

[17] Chaturvedi N, Stevens L, Fuller JH (1997). Which features of smoking determine mortality risk in former cigarette smokers with diabetes? The world health organization multinational study group. Diabetes Care.

[18] Valabhji J, Barron E, Bradley D (2020). Early outcomes from the English National health service diabetes prevention programme. Diabetes Care.

[19] Le Roux CW, Astrup A, Fujioka K (2017). 3 years of liraglutide versus placebo for type 2 diabetes risk reduction and weight management in individuals with prediabetes: a randomised, double-blind trial. The Lancet.

[20] Wilding JPH, Batterham RL, Calanna S (2021). Once-weekly semaglutide in adults with overweight or obesity. N Engl J Med.

[21] Excellence NIfCaC. NICE recommends new treatment option for adults with obesity and non-diabetic hyperglycaemia who have a high risk of cardiovascular disease | News and features | News | NICE: 2020. Available from: https://www.nice.org.uk/news/article/nice-recommends-new-treatment-option-for-adults-with-obesity-and-non-diabetic-hyperglycaemia- who-have-a-high-risk-of-cardiovascular-disease.

[22] Bycroft C, Freeman C, Petkova D (2018). The UK Biobank resource with deep phenotyping and genomic data. Nature.

[23] Elliott P, Peakman TC, UK Biobank (2008). The UK Biobank sample handling and storage protocol for the collection, processing and archiving of human blood and urine. Int J Epidemiol.

[24] Levey AS, Eckardt K-U, Tsukamoto Y (2005). Definition and classification of chronic kidney disease: a position statement from kidney disease: improving global outcomes (KDIGO). Kidney Int.

[25] Wise J (2014). UK survey confirms link between deprivation and smoking. BMJ.

[26] Kar D, Gillies C, Nath M, Khunti K, Davies M, Seidu S (2019).

[27] Ioakeimidis N, Dima I, Terentes-Printzios D (2020). Combined effect of cigarette smoking and prediabetes on structural and functional changes of large arteries. Eur Heart J.

[28] Canoy D, Wareham N, Luben R (2005). Cigarette smoking and fat distribution in 21,828 British men and women: a population-based study. obes.

[29] Kim JH, Shim KW, Yoon YS, Lee SY, Kim SS, Oh SW (2012). Cigarette smoking increases abdominal and visceral obesity but not overall fatness: an observational study. PLoS One.

[30] Lee S, Bacha F, Gungor N, Arslanian SA (2006). Waist circumference is an independent predictor of insulin resistance in black and white youths. J Pediatr.

[31] Bonnet F, Marre M, Halimi J-M (2006). Waist circumference and the metabolic syndrome predict the development of elevated albuminuria in non-diabetic subjects: the DESIR Study. J Hypertens.

[32] Pisinger C, Jorgensen T (2007). Waist circumference and weight following smoking cessation in a general population. Prev Med.

[33] Duncan MS, Freiberg MS, Greevy RA, Kundu S, Vasan RS, Tindle HA (2019). Association of smoking cessation with subsequent risk of cardiovascular disease. JAMA.

[34] Phisitkul K, Hegazy K, Chuahirun T (2008). Continued smoking exacerbates but cessation ameliorates progression of early type 2 diabetic nephropathy. Am J Med Sci.

[35] Campagna D, Alamo A, Di Pino A (2019). Smoking and diabetes: dangerous liaisons and confusing relationships. Diabetol Metab Syndr.

[36] Gæde P, Oellgaard J, Carstensen B (2016). Years of life gained by multifactorial intervention in patients with type 2 diabetes mellitus and microalbuminuria: 21 years follow-up on the Steno–2 randomised trial. Diabetologia.

[37] Chuahirun T, Wesson DE (2002). Cigarette smoking predicts faster progression of type 2 established diabetic nephropathy despite ACE inhibition. Am J Kidney Dis.

[38] Oakes JM, Fuchs RM, Gardner JD, Lazartigues E, Yue X (2018). Nicotine and the renin-angiotensin system. Am J Physiol-Regulatory, Integrat Comparat Physiol.

[39] Trap-Jensen J, Carlsen JE, Svendsen TL, Christensen NJ (1979). Cardiovascular and adrenergic effects of cigarette smoking during immediate non-selective and selective beta adrenoceptor blockade in humans. Eur J Clin Invest.

[40] Kar D, Gillies C, Zaccardi F (2016). Relationship of cardiometabolic parameters in non-smokers, current smokers, and quitters in diabetes: a systematic review and meta-analysis. Cardiovasc Diabetol.

[41] Molitch ME, Rupp D, Carnethon M (2006). Higher levels of HDL cholesterol are associated with a decreased likelihood of albuminuria in patients with long-standing type 1 diabetes. Diabetes Care.

[42] Shankar A, Klein R, Moss SE, Klein BEK, Wong TY (2004). The relationship between albuminuria and hypercholesterolemia. J Nephrol.

[43] Wang Y-X, Wang A-P, Ye Y-N (2019). Elevated triglycerides rather than other lipid parameters are associated with increased urinary albumin to creatinine ratio in the general population of China: a report from the REACTION study. Cardiovasc Diabetol.

[44] Ceriello A, De Cosmo S, Rossi MC (2017). Variability in HbA1c, blood pressure, lipid parameters and serum uric acid, and risk of development of chronic kidney disease in type 2 diabetes. Diabetes Obes Metab.

[45] Rotbain Curovic V, Theilade S, Winther SA (2021). Visit-to-visit variability of clinical risk markers in relation to long-term complications in type 1 diabetes. Diabet Med.

[46] Smoking prevalnce in the UK and the impact of data collection changes: 2020; 7th December 2021. Available from:https://www.ons.gov.uk/peoplepopulationandcommunity/healthandsocialcare/drugusealcoholandsmoking/bulletins/smokingprevalenceintheukandtheimpactofdatacollectionchanges/2020#:∼:text=Characteristics %20of%20current%20cigarette%20smokers,and%2014.0%25% 20 (around%203.3.

