# Supplementary material for: Predictors and determinants of albuminuria in people with prediabetes and diabetes based on smoking status: A cross-sectional study using the UK Biobank data
Source: eClinicalMedicine. 2022 Jul 2;51:101544. doi: 10.1016/j.eclinm.2022.101544 (PMC9256818; doi:10.1016/j.eclinm.2022.101544)
Supplement: Supplementary file 1 [file mmc1.docx]

***Supplementary material 1 – descriptiive statistics***

**Table 1: Relationship between DM status and albuminuria**

| glycaemic status | Normoalbuminuria | Albuminuria | Total | p-value |
| --- | --- | --- | --- | --- |
| Normoglycaemia | 98703 | 25163 | 123866 | <0.001 |
| Prediabetes | 6113 | 2740 | 8853 | <0.001 |
| Diabetes | 5646 | 4249 | 9895 | <0.001 |

**Table 2: Relationship between smoking status and albuminuria**

| Albuminuria status | current smoker | Ex-smoker | Non-smoker | Total | p-value |
| --- | --- | --- | --- | --- | --- |
| normoalbuminuria | 13831 | 41072 | 62701 | 117604 | <0.001 |
| albuminuria | 4844 | 12918 | 16599 | 34361 | <0.001 |
| total | 18675 | 53990 | 79300 | 151965 | <0.001 |

**Table 3: Townsend deprivation scale (-6.26 - least deprived, 11.00 - most deprived)**

| **Statistics** | | |
| --- | --- | --- |
| Townsend deprivation index at recruitment | | |
| Number | Valid | 501866 |
|  | Missing | 625 |
| Median | | -2.135 |
| Mode | | -4.697 |
| Minimum | | -6.258 |
| Maximum | | 11.001 |
| Percentiles | 20 | -3.933 |
|  | 25 | -3.639 |
|  | 40 | -2.764 |
|  | 50 | -2.135 |
|  | 60 | -1.295 |
|  | 75 | 0.550 |
|  | 80 | 1.3496 |

**Table 4: Investigation of missing value based on age (Age band 0 = <60; 1 = ≥ 60 years)**

**
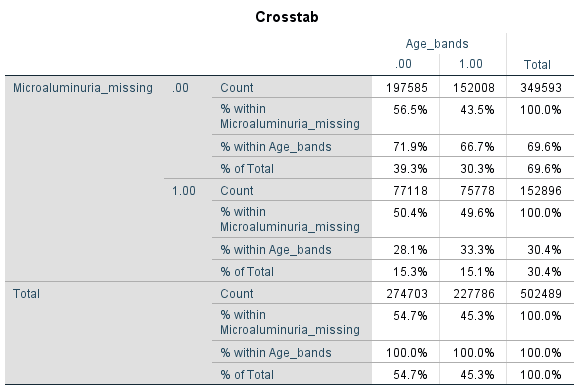
**

**Table 5: Investigation of missing value based on gender (0 – female, 1 – male)**

**
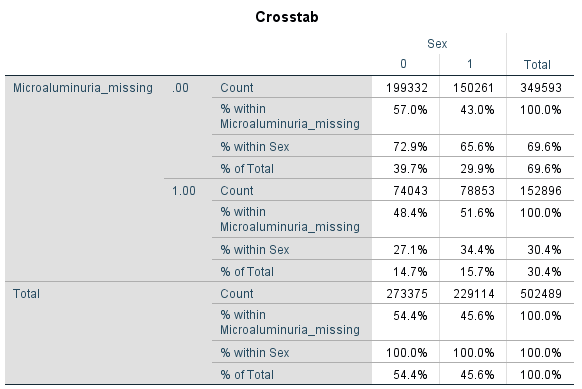
**

**Table 6: Smoking prevalence based on albuminuria and glycaemia status**

| Prediabetes | Smoking status | Normoalbuminuria (n) | Albuminuria  (n) | Total  (%) |
| --- | --- | --- | --- | --- |
|  | **Non-smoker** | 2575 | 1111 | 3686 (44.1%) |
|  | **Ex-smoker** | 2226 | 1120 | 3346 (40.0%) |
|  | **Smoker** | 868 | 463 | 1331 (15.9%) |
| Diabetes | **Non-smoker** | 2465 | 1815 | 4280 (43.8%) |
|  | **Ex-smoker** | 2244 | 1961 | 4205 (43.0%) |
|  | **Smoker** | 649 | 640 | 1289 (13.2%) |

**Table 7: Group statistics for mean value of cardiorenal risk factors based on glycaemic status**

| Cardiorenal risk factors | Glycaemic status | Number | Mean | Standard deviation | Standard error of mean |
| --- | --- | --- | --- | --- | --- |
| **Age** | Prediabetes | 20305 | 57.01 | 8.13 | 0.057 |
|  | Diabetes | 17609 | 57.10 | 8.09 | 0.061 |
| **SBP** | Prediabetes | 19030 | 144.79 | 19.48 | 0.141 |
|  | Diabetes | 16415 | 144.76 | 18.08 | 0.147 |
| **DBP** | Prediabetes | 19031 | 83.45 | 10.76 | 0.078 |
|  | Diabetes | 16416 | 82.54 | 10.81 | 0.084 |
| **HbA1c** | Prediabetes | 20305 | 35.42 | 4.09 | 0.028 |
|  | Diabetes | 17608 | 50.78 | 11.74 | 0.088 |
| **HDL** | Prediabetes | 17726 | 1.28 | 0.33 | 0.002 |
|  | Diabetes | 15304 | 1.19 | 0.33 | 0.003 |
| **LDL** | Prediabetes | 19307 | 3.34 | 0.97 | 0.007 |
|  | Diabetes | 16658 | 2.86 | 0.88 | 0.007 |
| **BMI** | Prediabetes | 20172 | 30.57 | 5.63 | 0.040 |
|  | Diabetes | 17428 | 31.69 | 5.91 | 0.045 |
| **UAC** | Prediabetes | 8853 | 44.36 | 183.21 | 1.947 |
|  | Diabetes | 9895 | 77.22 | 273.13 | 2.75 |
| **Cholesterol** | Prediabetes | 19352 | 5.35 | 1.29 | 0.009 |
|  | Diabetes | 16720 | 4.71 | 1.18 | 0.009 |

**Table 8: Tests to satisfy the assumption for logistic regression**

**
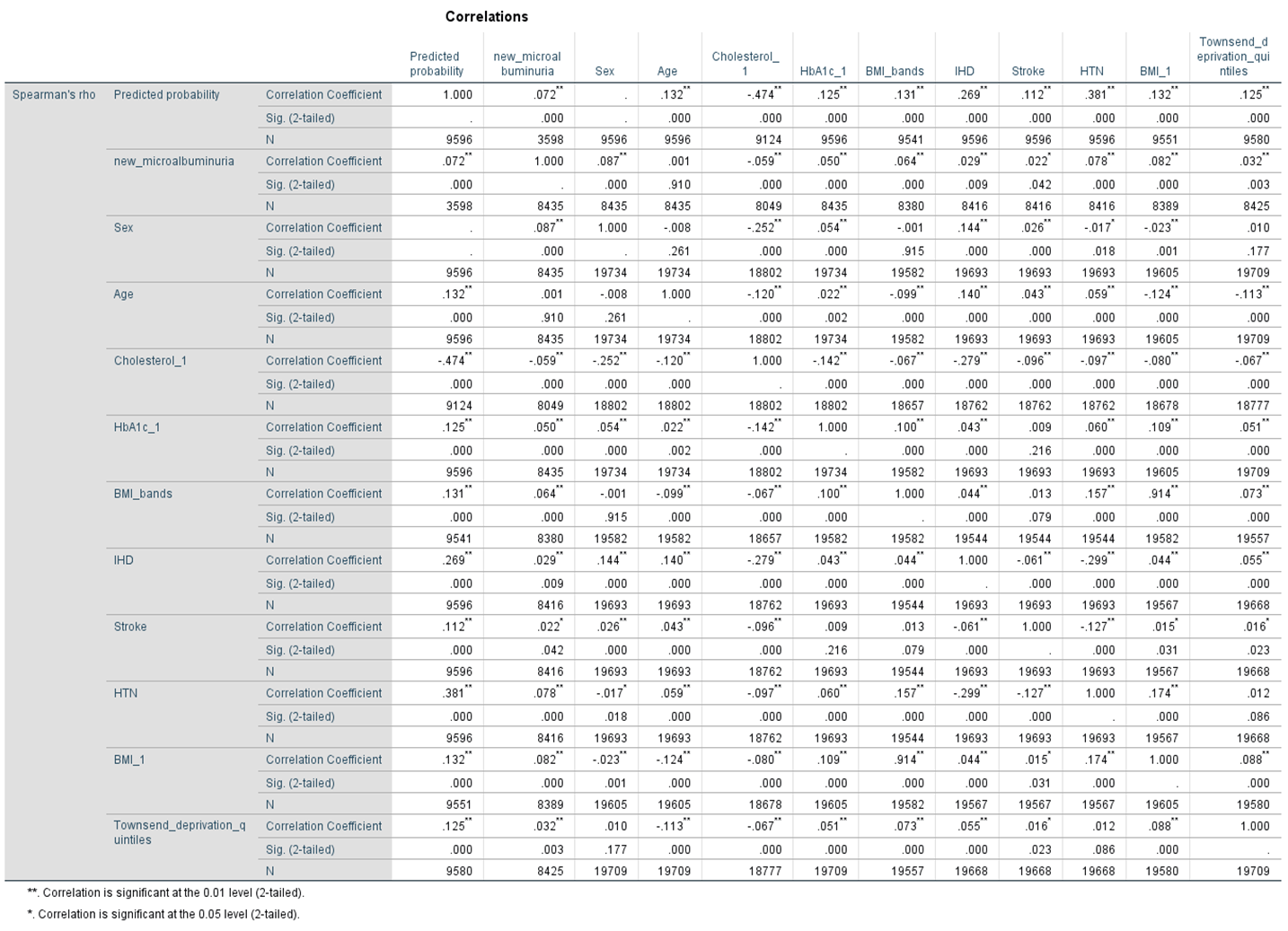
**

***Supplementary material 2 – distribution of data***


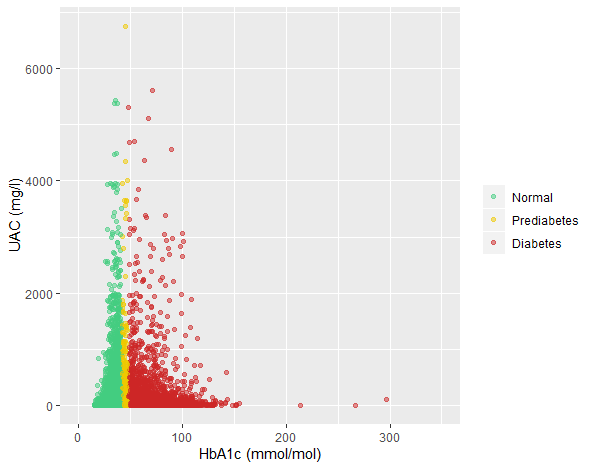


**Figure 1: Scatter plot showing the distribution of UAC in different glycaemic spectrum**

**
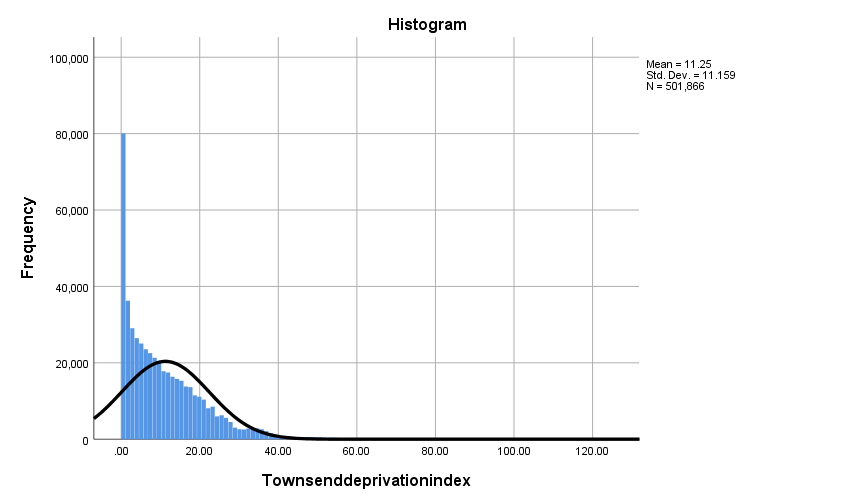
**

**Figure 2: Distribution of Townsend Deprivation Index data**

**Table 1: Tests for Normality**

**
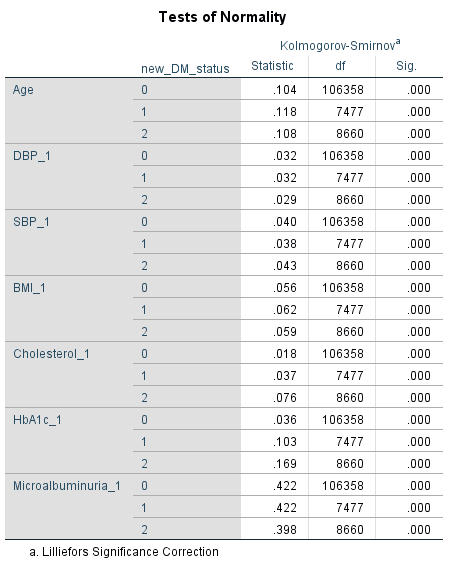
**

***Supplementary material 3 – correlation analyses***


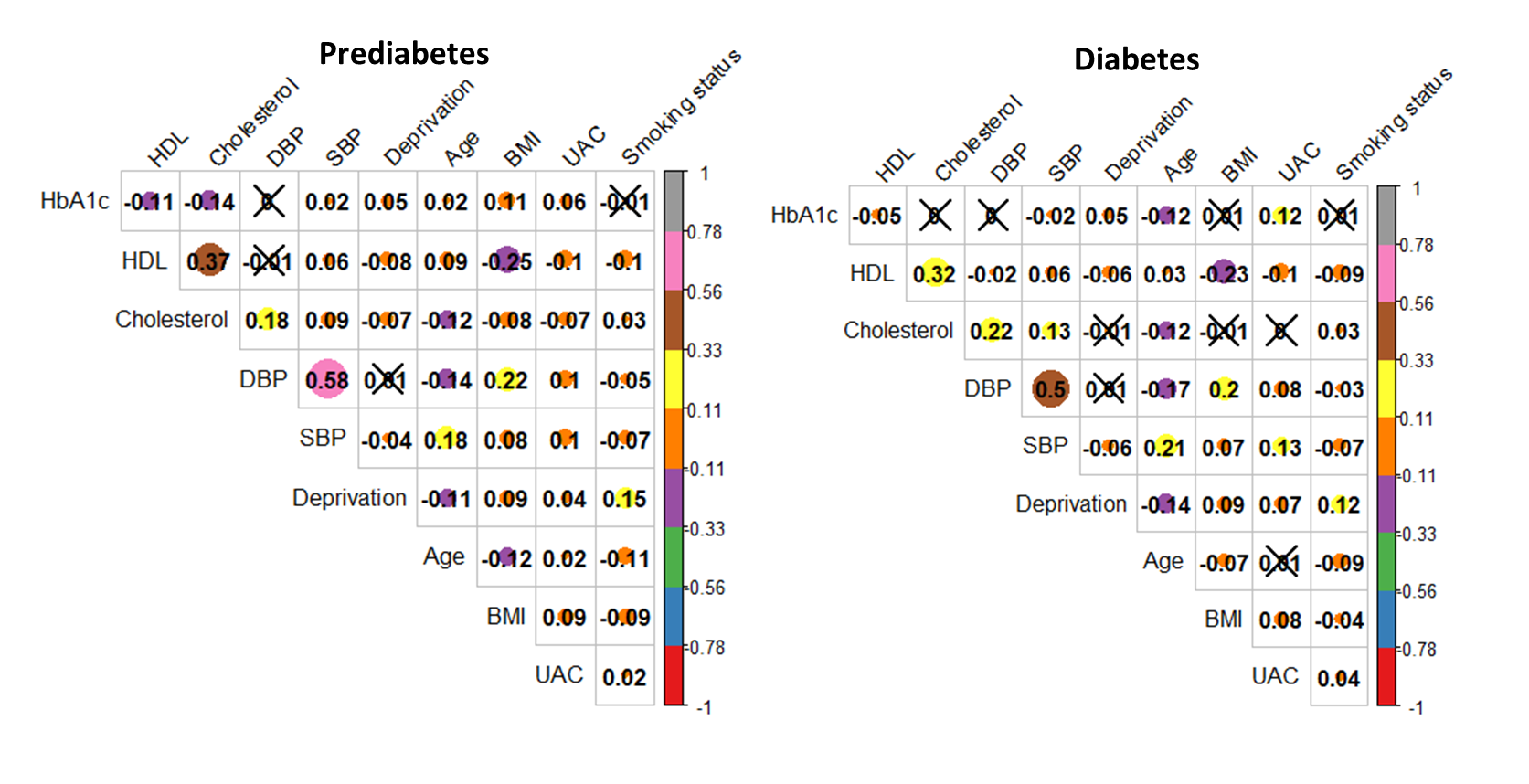


***Figure 1: Spearman correlation analysis of cardiorenal risk factors***
