# Supplementary material for: Predictors and determinants of albuminuria in people with prediabetes and diabetes based on smoking status: A cross-sectional study using the UK Biobank data
Source: eClinicalMedicine. 2022 Jul 2;51:101544. doi: 10.1016/j.eclinm.2022.101544 (PMC9256818; doi:10.1016/j.eclinm.2022.101544)
Supplement: Supplementary file 2 [file mmc2.docx]

**CAPTIONS FOR SUPPLEMENTARY MATERIALS**

1. Supplementary materials 1 – Descriptive Statistics
2. Table 1 – Relationship between DM status and albuminuria
3. Table 2 – Relationship between smoking status and albuminuria
4. Table 3 – Townsend Deprivation Scale
5. Table 4 – Investigation of missing value based on age
6. Table 5 - Investigation of missing value based on gender
7. Table 6 – Smoking prevalence based on albuminuria and glycaemic status
8. Table 7 – Group statistics for mean value of cardiorenal risk factors based on glycaemic status
9. Table 8 – Tests to satisfy the assumptions for logistic regression
10. Supplementary materials 2 – Distribution of Data
11. Figure 1: Scatter plot
12. Figure 2: Histogram – Townsend deprivation index showing non-parametric distribution
13. Table 1 – Tests for Normality
14. Supplementary materials 3 – Correlation analysis
15. Figure 1: Spearman’s ranked correlation
